# The Role of IL-6 in Cancer Cell Invasiveness and Metastasis—Overview and Therapeutic Opportunities

**DOI:** 10.3390/cells11223698

**Published:** 2022-11-21

**Authors:** Magdalena Rašková, Lukáš Lacina, Zdeněk Kejík, Anna Venhauerová, Markéta Skaličková, Michal Kolář, Milan Jakubek, Daniel Rosel, Karel Smetana, Jan Brábek

**Affiliations:** 1 Department of Cell Biology, Faculty of Science, Charles University, 120 00 Prague, Czech Republic; 2 BIOCEV, Faculty of Science, Charles University, 252 50 Vestec, Czech Republic; 3 Centre for Tumour Ecology, First Faculty of Medicine, Charles University, 120 00 Prague, Czech Republic; 4 BIOCEV, First Faculty of Medicine, Charles University, 252 50 Vestec, Czech Republic; 5 Department of Paediatrics and Adolescent Medicine, First Faculty of Medicine, Charles University and General University Hospital, 120 00 Prague, Czech Republic; 6 Institute of Anatomy, First Faculty of Medicine, Charles University, 120 00 Prague, Czech Republic; 7 Department of Dermatovenereology, First Faculty of Medicine, Charles University and General University Hospital, 120 00 Prague, Czech Republic; 8 Laboratory of Genomics and Bioinformatics, Institute of Molecular Genetics, Czech Academy of Sciences, 140 00 Prague, Czech Republic

**Keywords:** IL-6, cancer, metastasis

## Abstract

Interleukin 6 (IL-6) belongs to a broad class of cytokines involved in the regulation of various homeostatic and pathological processes. These activities range from regulating embryonic development, wound healing and ageing, inflammation, and immunity, including COVID-19. In this review, we summarise the role of IL-6 signalling pathways in cancer biology, with particular emphasis on cancer cell invasiveness and metastasis formation. Targeting principal components of IL-6 signalling (e.g., IL-6Rs, gp130, STAT3, NF-κB) is an intensively studied approach in preclinical cancer research. It is of significant translational potential; numerous studies strongly imply the remarkable potential of IL-6 signalling inhibitors, especially in metastasis suppression.

## 1. Introduction

Interleukin 6 (IL-6) belongs to a broad class of small proteins involved in the regulation of various homeostatic and pathological processes, including embryonic development, wound healing and ageing, inflammation, and immunity, including COVID-19 [1].

Specifically, IL-6 is classified to be a part of the IL-6 family group of cytokines whose receptor complexes associate with either two (IL-6 and IL-11) or one (the rest of the cytokines) glycoprotein 130 (gp130) subunits [2]. Additional cytokines belonging to the family include IL-6 itself, IL-11, ciliary neurotrophic factor, cardiotrophin 1, cardiotrophin-like cytokine, leukaemia inhibitory factor, oncostatin M, and IL-27 [3,4].

**Figure 1:**
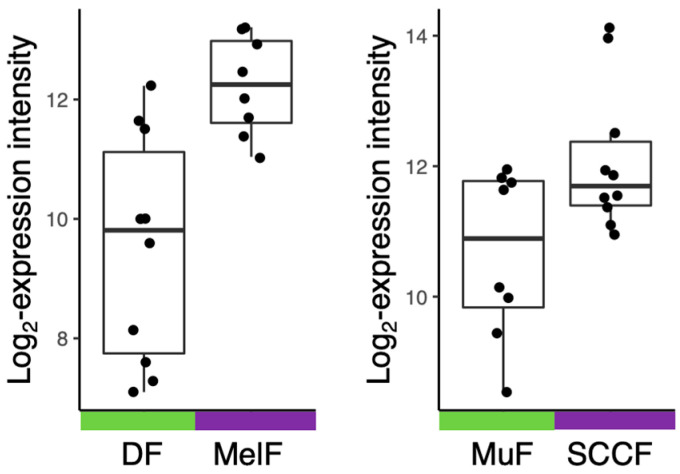
Expression of the *IL6* gene in stromal elements of melanoma and head and neck squamous cell carcinoma (HNSCC). Normal dermal fibroblasts (DF, (**left panel**)) and normal mucous fibroblasts (MuF, (**right panel**)) express lower quantities of IL-6 mRNA than cancer-associated fibroblasts isolated from melanoma (MelF, (**left panel**)) or HNSCC (SCCF, (**right panel**)).

**Figure 2:**
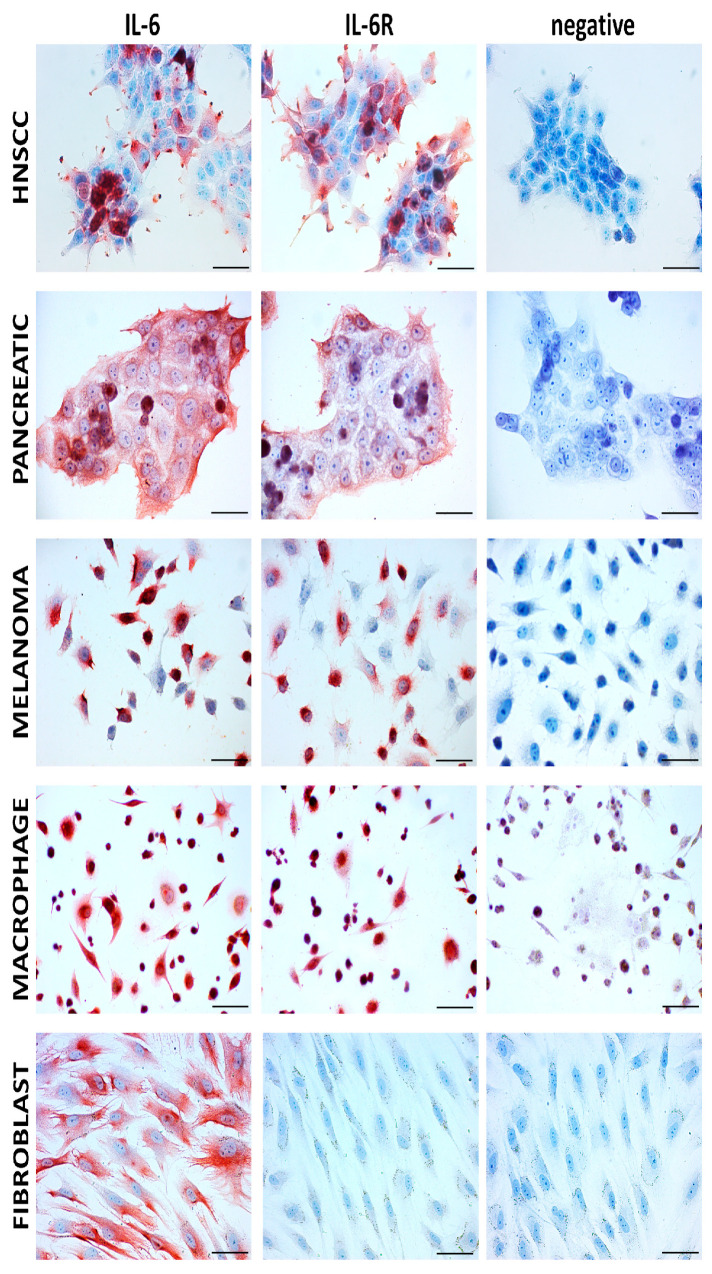
Immunocytochemical analysis of IL-6 and IL-6 receptor in cell lines representing the malignant (several types) and the stromal component of tumours using immunoperoxidase reaction; positive staining is visualised by red AEC (3-amino-9-ethylcarbazole) substrate deposition. HNSCC–head and neck squamous cell carcinoma–cell line FaDu (CVCL_1218), pancreatic ductal carcinoma cell line PaTu n (CVCL_1846), melanoma cell line BLM (CVCL_7035). Macrophages were obtained by the standard protocol using the THP-1 monocytic cell line (CVCL_0006). Fibroblasts represented here are primary human isolates of dermal origin. Negative control was performed using isotype control. Gill’s haematoxylin (blue) was used for counterstaining. The bar represents 100 μm.

IL-6 was initially identified in 1986 by Hirano et al. as a pro-inflammatory cytokine produced by immune cells and since then has been implicated in a wide variety of pathologies ranging from chronic inflammatory conditions to cancer [5]. Currently, it is understood from numerous reviews that IL-6 production is not limited exclusively to immune cells but can also be synthesised by parenchymal cells of the skin, intestinal tract, smooth muscle, lung tissue, or stroll cells such as mesenchymal cells and fibroblasts [3,6]. Release of IL-6 from these stromal cells, such as fibroblasts, is depicted in Figure 1 based on Novák et al. [7] and the author’s unpublished data. Pathologically, IL-6 is produced by tumour stromal cells, immune cells, trafficking to the cancerous lesion, or the cancer cells themselves (Figure 2). Sources of IL-6 do not have to be limited to the tumour microenvironment (TME) but can also be produced by hematopoietic stem and progenitor cells (HPSC), epithelial cells, or muscle tissue, all contributing to the rigorous inter-cellular cross-talk, vital for the advancement of the disease [8,9]. This aberrant activation of downstream IL-6 signalling pathways is associated clinically and experimental with poor outcomes in oncological patients or cancer models [8,10]. Therefore, what is of particular interest in the area of cancer research are the effects of IL-6 on both stromal and parenchymal cells in promoting the invasiveness of the tumour and its ensuing metastasis.

Given that, IL-6 is of particular interest in cancer research. In this review, we aimed to summarise the effects of IL-6 on both stromal and parenchymal cells, particularly on promoting tumour invasiveness and thus ensuing metastasis.

## 2. IL-6 Signalling and Downstream Effects

IL-6 signalling can follow either the classical or the trans-signalling pathway. Whereas the classical pathway is vital in the acute-phase immune response, regeneration, and haematopoiesis, the trans pathway allows cells not expressing the IL-6 receptor (IL-6R) to become responsive to this signal and initiate downstream signalling of IL-6 [3,10,11]. This allows such cells to engage in response to IL-6 stimuli and to become active participants rather than bystanders.

The classical IL-6 signalling pathway is initiated by IL-6 binding to a membrane-bound specific receptor, IL-6R. The ligand/receptor complex then associates with membrane-spanning gp130, resulting in the formation of a trimeric complex. Consequently, by further dimerisation, a heterohexameric signal-transducing receptor complex arises [5,11,12,13]. While IL-6R is limited in its expression to neutrophils, hepatocytes, monocytes/macrophages, and some lymphocytes, gp130 is ubiquitously expressed in most cell types [4,5,11,12]. However, cells that express gp130 alone are unable to bind IL-6 and are, therefore, not responsive to its effects. This highlights the relevance of the alternative *trans* pathway [2].

The *trans*-signalling pathway was discovered as a consequence of the detection of soluble IL-6R (sIL-6R) in human serum and urine samples [13]. The presence of sIL-6R and other cytokine receptors in body fluids is a general phenomenon that occurs under physiological conditions [13]. It was later confirmed that sIL-6R was markedly increased in several inflammatory diseases, such as chronic inflammatory bowel disease (in the serum) and rheumatoid arthritis (in the serum and synovial fluid). Of note, sIL-6R can promote tumorigenesis in cancers linked to long-standing inflammation, such as colitis-associated cancer [4,10,14,15,16,17,18]. sIL-6R is generated by cleavage via metalloproteases ADAM10 and ADAM17 and is shed from the membrane. It can bind circulating IL-6 and then form the necessary trimeric complex with gp130. The complex formation is followed by dimerisation and activation of downstream signalling [10,12,14]. It is of interest that, to a lesser extent, sIL-6R can also be generated via alternative splicing of pre-mRNA [19].

The trans pathway is critical in the context of cancer because it influences tumour and surrounding stromal cells that do not express IL-6R, thus modifying the activity and recruitment of cells into the TME [5,11]. For example, direct stimulation of tumour cells via IL-6 can induce increased proliferation and invasiveness. Paracrine or autocrine IL-6 signalling prompts stromal and immune cells to secrete additional signalling molecules such as VEGF for angiogenesis or pro-inflammatory cytokine IL-1β [10]. Thus, IL-6-initiated signalling gains higher complexity and involves multifaceted mechanisms of action crucial for shaping the course of cancer progression.

Regardless of the mechanism of IL-6/IL-6R/gp130 complex formation, it uniformly leads to the recruitment of Janus kinases (JAKs). JAKs, in turn, provide protein docking sites for additional pro-proliferative, pro-survival signalling pathways such as JAK/signal transducer and activator of transcription (STAT), PI3K/AKT, or for the RAS/RAF/MEK/MAPK pathways [5,10,12]. However, there are notable differences in downstream effects. In the classical pathway activation, the effects are associated with regenerative and anti-inflammatory results, as shown in several studies in murine models post partial hepatectomy [11,20]. On the other hand, it is the pro-inflammatory effect of the *trans* pathway activation that is implicated in the TME [10,11].

In order to emphasise the relevance of IL-6 downstream signalling and its role in cancer, we will primarily focus on the effects of the JAK/STAT3 signal transduction pathway, which has the ability to module tumour cell proliferation, survival, invasion, and metastasis, and thus is strongly associated with the progression of malignant disease. Following heterohexameric signal-transducing receptor complex formation, kinases JAK1, JAK2, and tyrosine kinase 2 (TYK2) associate with gp130. These kinases undergo activation via reciprocal transphosphorylation, thus allowing phosphorylation of tyrosine residues in the cytoplasmic region of gp130. Phosphorylated gp130 will now be able to interact with STAT3. Due to the proximity of STAT3 to activated JAKs, STAT3 is also activated via phosphorylation. Activated STAT3 forms a homodimer and acts as a transcription factor. In the nucleus, activated STAT3 targets regulatory sequences of genes encoding pro-proliferation factors such as cyclin-D1 and cMYC, pro-survival factors such as Bcl-XL and Bcl-2, and pro-angiogenic factors such as VEGF [10,11,14,21]. Increased STAT3 signalling and upregulated levels of cyclin-D1 and cMYC expedite progression through the cell cycle, while pro-survival factors also suppress apoptosis in cancer cells [22]. Furthermore, IL-6 downstream effects also modulate the activity of neutrophils, natural killer cells, or T cells, resulting in a decreased immune response to the neoplasm, despite the apparent trafficking of these immune cells to the lesion. This mechanism allows for the development of an immune tolerance [10,23]. IL-6 simultaneously upregulates T regulatory cells and myeloid-derived suppressor cells. Their activation further contributes to the remarkably immunosuppressed TME, resulting in a severely impaired anti-tumour immune response.

Thus, it becomes increasingly apparent how versatile IL-6 signalling is. In cancer, via stimulation of proliferation, survival, angiogenesis, or evasion of immune detection, it potentiates and propagates pro-cancerogenic signals within the TME. Further discussion will concentrate on the effects of IL-6 in the invasion-metastasis cascade across a range of cancer types, showing the applicability of targeting both IL-6 signalling and tumour cell migration as a therapeutic goal in cancer treatment.

## 3. IL-6 Signalling in Promoting Tumorigenesis, Invasiveness, and Metastasis in Cancer

IL-6 has been identified as a cytokine abundantly present in the TME of various tumour types, including head and neck squamous cell carcinoma (HNSCC) [24,25,26,27], pancreatic cancer [28,29], non-small-cell lung cancer [30], breast cancer [14,31], ovarian cancer [19,32], and melanoma [33,34]. In addition to being relevant in the course of tumorigenesis, IL-6 also facilitates the series of events that must occur as a prerequisite for the formation of a secondary tumour, a metastasis.

Investigation of the isolated mechanisms contributing to cancer progression in individual tissues and cancer types is extremely valuable. However, a unifying hallmark across nearly all types of solid tumours that also accounts for the highest mortality is the process of metastatic spread. As such, it merits in-depth investigation [31,35]. Metastasis formation is understood as a series of events beginning with (i) localised migration and invasion of the surrounding extracellular matrix (ECM), (ii) intravasation into nearby vessels, (iii) survival in extreme conditions in the bloodstream or lymphatic vessels, (iv) extravasation into the parenchyma of the tissue, and finally (v) modification of the activity of tumour cells to allow them to thrive in a new environment [36,37,38]. Just as metastasis formation is a common process occurring in a variety of types of cancer, aberrant IL-6 signalling provides another unifying motif that supports tumour growth and metastasis. Therefore, it provides IL-6 with a position as a promising therapeutic target. To demonstrate just how consequential this cytokine is, the metastatic cascade will be discussed in light of IL-6 signalling in selected cancer types.

## 4. IL-6 Contributes to the Formation of the Pre-Metastatic Niche

Early observations in breast cancer led Stephen Paget to coin his “seed” (cancer cells) and “soil” (host tissue) hypothesis of metastasis. In these pioneer times, Paget scrutinised why and how some organs were affected by metastases while others remained unscathed [39]. Only more recently, sufficient data were gathered to shed light on this process mediated by tumour-secreted factors, extracellular vesicles (EV), and bone marrow-derived cells (BMDCs). All these tightly orchestrated mechanisms prepare the preselected pre-metastatic niche (PMN) for the arrival of cancer cells [9,40,41,42]. The sequential preparation of the PMN involves changes such as vascular leakiness, allowing CSC extravasation and activation of stromal cells and restructuring of the extracellular matrix (ECM), facilitating recruitment of cell types such as BMDCs for PMN formation [41,42]. The most important cellular component of the PMN is BMDCs, especially immune cells, all derived from haematopoietic progenitor and stem cells (HPSCs) [9,42]. HPSCs can respond to conditions such as injury and inflammation or to tumour-secreted stimuli, promoting their differentiation [9,42]. Perhaps the most significant population of differentiated HPSCs are myeloid-derived suppressor cells (MDSCs). MDSCs maintain a chronic pro-inflammatory and yet immunosuppressed environment within the PMN [9,43]. It is unclear how and why the MDSCs migrate to the PMN. However, data show that a number of soluble factors, including IL-6, may regulate MDSC recruitment, activation, and differentiation within the PMN [Talmadge history].

Magidey-Klein et al. used paired breast cancer or melanoma cell lines, one with a high frequency of metastasis (met-high) and one with a low frequency of metastasis (met-low), to study the role of IL-6 in HPSC differentiation, metastasis, and its involvement in the generation of the PMN. Cells were transplanted into mouse models with comparable success and presented similar tumour growth between the met-high and met-low groups. However, lung metastases were significantly more frequent in the high-met group [9]. When analysing the cellular composition of the bone marrow, mice with tumours were also observed to have a higher percentage of LSK cells (considered as HPSCs) than control mice. Specifically, the monocyte dendritic progenitor (MDP) population was elevated in both cancer types when compared to other progenitor cell types. Overall, the results showed that met-high tumours induce myeloid-biased differentiation of HPSCs, which correlates with tumour aggressiveness and metastatic potential. IL-6 was identified as a mediator for the cross-talk between bone marrow and cancer cells, and the levels of IL-6 correlated with MDP growth and increased incidence of metastasis. In met-low melanoma cells, overexpression of IL-6 was a sufficient signal to educate MDPs to induce metastasis and metastatic switch. MDPs further differentiated into M2 pro-inflammatory and immunosuppressive macrophages, localised at the metastatic sites. Magidey-Klein and co-workers put forward a new role of tumour-derived IL-6 in driving the differentiation of HPSCs toward pro-metastatic MDPs. This shows the importance of IL-6 not only in the context of TME but also in its potential to orchestrate the bone marrow niche, which is vital for the eventual formation of the PMN.

**Figure 3:**
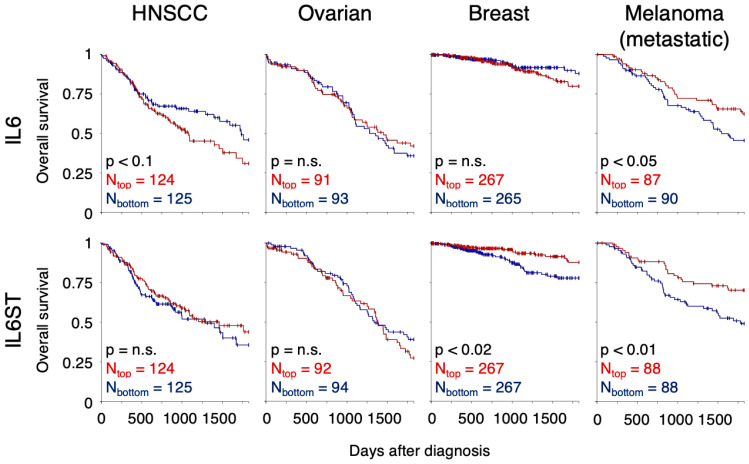
Correlation of patient survival with mRNA expression of IL-6 pathway components. Overall survival of patients suffering from HNSCC, ovarian cancer, breast cancer, or metastatic melanoma is evaluated for patients with different levels of IL6 mRNA expression (**top**) and IL6ST mRNA expression (**bottom**). Survival of the patients with the highest gene expression (4th quartile, N_top_ patients) was compared with the survival of the patients with the lowest expression (1st quartile, N_bottom_) using Kaplan–Meier curves and the log-rank test. The analysis was performed within the Xena platform [44].

The influence of mRNA expression of the IL-6 signalling components on patient survival is, however, not direct. While in HNSCC, low expression of IL6 mRNA improves survival of the patients with marginal statistical significance (Figure 3, XENA [44]), in ovarian and breast cancers, there is no difference in survival of the patients with high IL6 expression. In metastatic melanoma, low expression of IL6 shortens the patient survival, paradoxically. High expression of IL6ST, the gene coding for gp130, significantly improves the survival of patients suffering from breast cancer and metastatic melanoma (Figure 3). No such correlation was observed for IL6R mRNA expression.

## 5. Metastasis Repression by Targeting IL-6 Signalling

**Figure 4:**
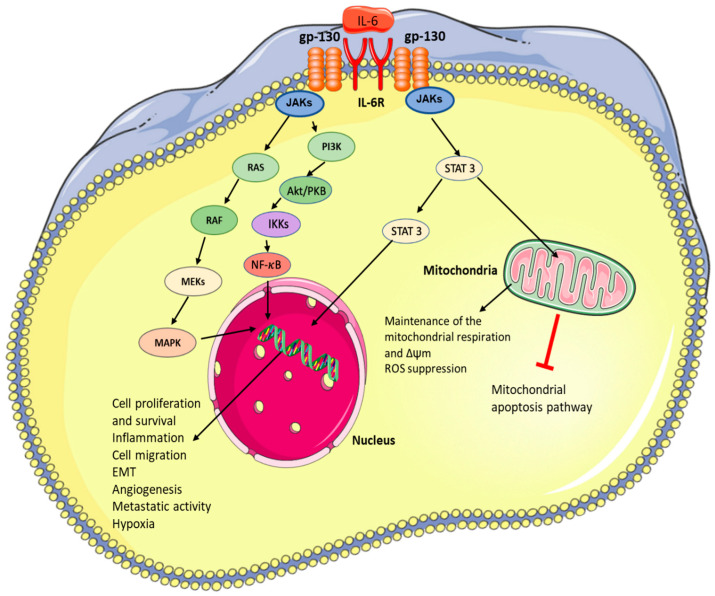
Influence of IL-6 signalling on cancer development and metastasis formation. The figure was created using Servier Medical Art available at http://smart.servier.com/ (accessed on 15 August 2022).

As a result of the ubiquitousness of IL-6 pro-cancerogenic signalling, the molecule presents itself as a therapeutic target that will “pack a punch”. IL-6 signalling is associated with increased invasiveness, aggressiveness, and incidence of metastasis across many tumours (Figure 4) [3,8,45,46].

**Figure 5:**
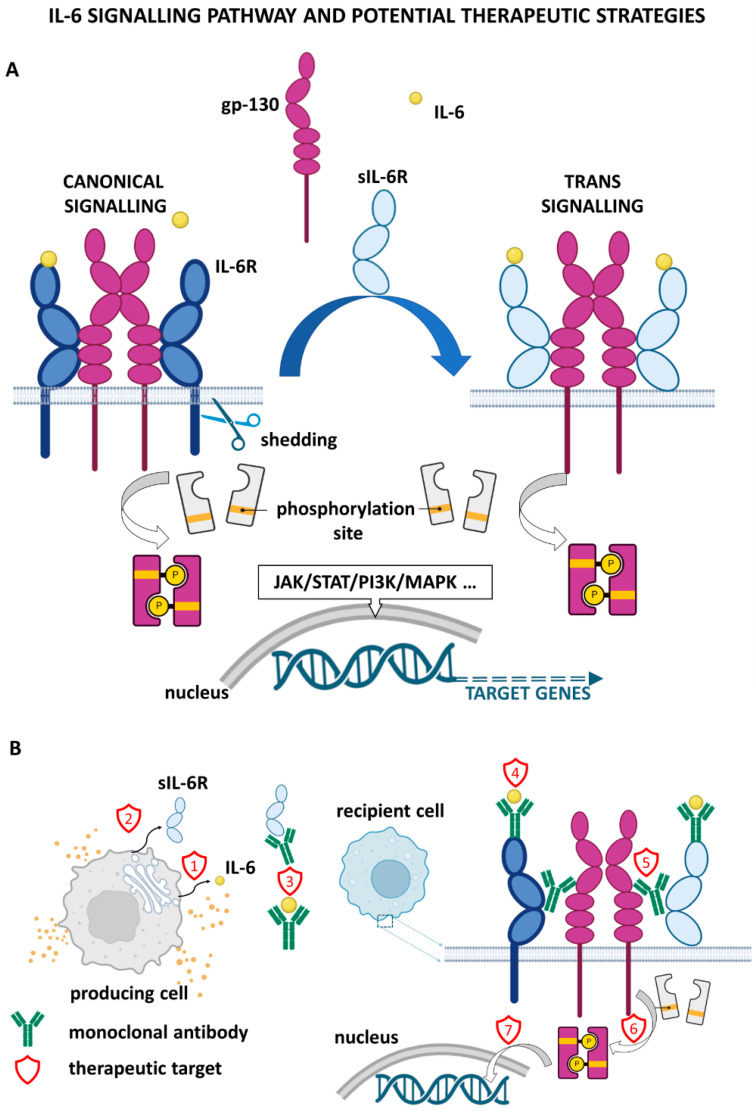
Therapeutic strategies for the inhibition of IL-6 signalling.(**A**) intracellular signaling, (**B**)intercellular signaling. (1) cytokine synthesis/release blockade, (2) sIL-6R shedding blockade, (3) cytokine/soluble receptor neutralizing, (4) cytokine binding to receptor prevention, (5) heterotetrameric complex formation blockade, (6) signal transducer kinase activity inhibition, (7) downstream signalling blockade/gene transcription blockade.

Recent data also implicate this protein in preparation for the PMN, which is another essential step prior to cancer cell dissemination. Therefore, since IL-6 plays a central role in the invasion-metastasis cascade, which is the leading cause of cancer-related deaths worldwide, it is an absolutely obligatory avenue for novel pharmacological interventions [3,8,45,46]. Inhibition of IL-6 and its signalling pathways is an intensively studied therapeutic approach in cancer treatment. Strategies explore general inhibition of the IL-6 signalling axis (IL-6/6IL-6R/gp130), including downstream signalling proteins such as STAT3, NF-κB, and HIF-1α. The most frequently used/studied strategies for the inhibition of IL-6 signalling are shown in Figure 5, and examples of possible therapeutic agents for targeting IL-6 and its pathway are summarised in Table 1.

Currently, the clinical and/or research applications of IL-6R targeting antibodies are utilised in a variety of fields. Tocilizumab, an IL-6R antibody, is clinically used in the treatment of various autoimmune diseases, such as rheumatoid arthritis, which is associated with pathologically hyper-activated IL-6 signalling [47]. Despite the practical applications in the field of rheumatology, the therapeutic use of such biological treatments in oncology still requires optimisation. However, there are some encouraging data supporting this concept.

In the case of recurrent ovarian carcinoma, tocilizumab decreased STAT3 activation/phosphorylation in patient immune cells (e.g., myeloid cells, CD4+ T and CD8+ T only at a high dose), most probably due to the suppression of IL-6R signalling [88]. It suggests that tocilizumab could suppress IL-6-induced immunosuppression (e.g., induction of macrophage M2 phenotype and Treg attraction) [89,90]. However, patients with acute leukaemia or myelodysplasia did not show any improvements in long-term survival on tocilizumab treatment [91]. A combination of Sarilumab (antibody targeting soluble and membrane IL-6R; FDA-approved for rheumatoid arthritis) and Capecitabine is currently tested in a clinical trial (EMPOWER; NCT04333706) in triple-negative breast cancer patients (stage I-III, high-risk residual disease). Moreover, Nguyen et al. reported that siltuximab (IL-6R antibody) could repress the Wnt/β-catenin pathway [92].

An alternative strategy employs small molecular inhibitors of IL-6/gp130 signalling rather than IL-6R inhibitors [3]. This approach also shows potential for a beneficial anti-metastatic effect. Bazedoxifene (repurposed selective oestrogen receptor modulator) displays a strong inhibitory effect on gp130 (receptor kinase of IL-6R). In the case of cervical cancer cells (SiHa, HeLa, CaSki), bazedoxifene treatment leads to a decrease in cell migration and invasion and additionally decreases Siha tumour burden in mouse models [52]. Its effect is associated with a reduction of IL-6-induced GP130, STAT3 and ERK1/2 phosphorylation. Possible agents for inhibition of IL-6 signalling could also be based on the structural motif of madindolines (gp130 inhibition) [93] and bufadienolide (blocking interaction of IL-6R with IL-6 or gp130) [61,62].

STAT3 plays a key role in IL-6 signalling and carcinogenesis. STAT3 (respective STAT3α isoform) [94,95] is involved in the EMT and self-renewal of cancer stem cells, promoting metastasis and invasion. Its downstream effects are crucial for the formation of an immunosuppressive TME [10]. Specifically, in the context of TME, active STAT3 signalling induces repression of neutrophils, natural killer (NK) cells, effector T cells, and dendritic cells (DCs) and activates regulatory T (Treg) cells and MDSC populations. This immunosuppressed landscape is thought to contribute to the weakened ability of the immune system to respond to developing cancer. Despite STAT3′s critical contribution to forming a cancer-supportive TME, no drug targeting STAT3 itself has been clinically approved for this purpose until now. Nevertheless, several promising compounds are being intensively studied [96,97]. For example, Stattic (low molecular direct inhibitor of Src homology 2 domain) significantly represses STAT3 activation and expression in a murine orthotopic xenograft model of HNSCC. This effect is associated with a reduction of STAT3-mediated HIF-1α expression and tumour progression [64]. Similarly, the application of OPB-31121 (oral STAT3 inhibitor) repressed constitutive and IL-6-induced JAK/STAT3 signalling in gastric cancer lines [98]. In this cohort of patients with advanced colon and rectal tumours, OPB-31121 treatment was associated with tumour shrinkage. The application was safe and relatively well tolerated [66]. Nevertheless, its pharmacokinetics displays significant variability, and the mean blood C_max_ (1.19–12.9 ng/mL) was much lower than in this case of the mouse tumour xenograft model [65,66].

In the past, when targeting particular signalling cascades, it has proven to be both important and effective to target the pathway at all levels, e.g., receptor, second messenger, and subsequent signalling proteins. Therefore, another therapeutic opportunity is emerging at the level of gene expression since IL-6 cascade-activated STAT3 acts as a transcription factor. By inhibiting STAT3′s DNA binding ability, its capacity to act as a transcription factor would be eliminated. While galiellalactone inhibits STAT3 signalling by binding to its DNA-binding region, STAT3 phosphorylation/activation is not repressed [67,99]. In prostate cancer cell lines (LNCap DuCaP and VCaP), galiellalactone strongly repressed IL-6-induced activity of the androgen receptor (AR; e.g., PSA expression) [67]. In these primary tissue slice cultures from radical prostatectomy samples, reduced expression of AR-controlled genes (e.g., PSA, TMPRSS2 and FKBP5) was also observed. AR plays one of the key roles in the development of prostate cancer, and inhibition of its signalling has been a main therapeutic option to manage locally advanced and metastatic prostate cancer in clinics [100].

While cytosolic STAT3 is an obvious choice as a therapeutic target, it was shown that the dysfunction of mitochondrial STAT3 can create sufficient stress for the cancer cells to induce apoptosis. Therefore, a combination of mitochondria-targeting drugs (e.g., arsenic trioxide, tamoxifen, hydrocortisone and others) [101] with IL-6 signalling inhibitors could be a base for another attractive alternative. Mitochondrial STAT3 plays an important role in the control of cellular respiration in the mitochondria (e.g., complexes I and II of the electron transport chain) [102,103] and can strongly support the oncogenic process [104,105]. Over the last years, it was reported that the phenotype of metastatic cancer is associated with active mitochondrial oxidative phosphorylation. This plays a central role in the generation of ROS, cell death, survival, and metastasis [106,107]. In detail, metastatic cells’ survival in the blood and their homing to the metastatic site may depend on the mitochondrial oxidative phosphorylation [108,109]. The redox balance is regulated by very high glucose uptake, and the stimulated citrate cycle enhances mitochondrial membrane potential. Cancer cells can also capture glycolytic lactate produced by fibroblast, tumour, and stromal cells [110,111]. The obtained lactate is converted to pyruvate, and in the mitochondria, it provides electrons for the mitochondrial electron transport chains and energy for ATP production. This process is called the “reverse Warburg” effect.

Targeting STAT3 could be an effective way to repress the mitochondrial function in cancer cells. OPB-51602 (an SH2 domain-targeting STAT3 inhibitor) stimulates the formation of proteotoxic STAT3 aggregates and resulting mitochondrial dysfunction [112]. OPB-51602-related cytotoxicity was induced by glucose starvation and increased reliance of prostate cancer cells DU 145 on mitochondrial function. Similarly, a decrease in IL-6-induced STAT3 mitochondrial localisation leads to mitochondrial oxidative stress, loss of mitochondrial membrane potential, and subsequent apoptosis of cancer cells [113].

Repression of NF-κB activity could also be a promising approach to the inhibition of IL-6 signalling. NF-κB proteins are a group of oncogenic factors that, in turn, control the expression of pro-inflammatory signalling proteins, such as IL-6. There is increasing evidence suggesting that NF-κB targeting could also increase tumour sensitivity to the therapy (e.g., chemo- and radiotherapy) and delay/repress the loss of therapeutic effectiveness. Chemotherapeutics (e.g., paclitaxel, 5-fluorouracil or doxorubicin) can stimulate strongly increased production of cytokines such as IL-1β, IL-6, IL-8, CSF2, and CCL2 [106,114]. IL-6 activates NF-κB (via STAT3/AKT pathway) [115,116], which subsequently promotes the production and secretion of more cytokines [114].

In TME, IL-6 signalling can significantly enhance the hypoxic phenotype [117]. Chronic inflammation stimulates cycling hypoxia as a result of limited oxygen diffusion and increases consumption of oxygen by filtrating immune and hyperproliferating cancer cells, leading to the lack of oxygen in TME [118,119,120]. A hypoxic phenotype is associated with EMT transition and resistance to therapy, especially immunotherapy. For example, macrophage-derived IL-6 promotes EMT in primary hepatocellular carcinoma cells under hypoxic versus oxygenated conditions [117]. Notably, this EMT can be strongly repressed by tocilizumab. The increased levels of intracellular ROS produced during hypoxia lead to the stabilisation of HIF-1α and NF-κB. HIF-1α induces an immunosuppressive tumour microenvironment by recruiting Tregs, MDSCs and macrophages. The NF-κB signalling pathway can also support the production of inflammatory factors and the recruitment of inflammatory immune cells [119]. Finally, higher levels/activity of infiltrated inflammatory cells result in a repeated hypoxia cycle. It suggests that targeting the intratumoral inflammatory mechanism by targeting IL-6 signalling could repress the hypoxic phenotype. Nevertheless, hypoxia can stimulate the activity of IL-6 signalling and thereby potentially decrease the effectiveness of this therapeutic strategy.

## 6. Inhibition of IL-6 Signalling in Combination Therapy

It is well known that oncological diseases display significant heterogeneity and interindividual variability. Therefore, general oncogenic signalling pathways may be at least partially substitutable targets, and their simultaneous modulation can synergically abrogate tumorigenesis. For example, both IL-6 and IL-8 can activate STAT3 signalling via JAK 2 and significantly increase cell migration when the signalling occurs concurrently, as opposed to stimulation by IL-6 or IL-8 alone [121]. A possible approach to this corroborative effect could be their simultaneous dual targeting. Simultaneous inhibition of IL-6 and IL-8 receptors via Tocilizumab and Reparixin (inhibitor of C-X-C motif chemokine receptor 1) significantly decreased the expression of matrix metalloprotease in mouse MDA-MB-231 breast cancer model models and decreased the incidence of liver and lung metastasis [122]. Similarly, a combination of bazedoxifene and SCH527123 (inhibitor of C-X-C motif chemokine receptors; Il-8 receptors) synergically repressed STAT3 and Akt phosphorylation in ovarian cancer cells (OVCAR3, SKOV3, and CAOV3) and in mice bearing CAOV3 tumours when compared to agents inhibiting just IL-6 or IL-8 [123]. According to the proposed model, in this case, the effect of combination therapy on tumour growth was sometimes smaller compared to the application of single agents. In this connection, it is interesting to note that bazedoxifene could repress TNF-α activation of CD40 receptors and subsequent activation of the NF-κB, STAT3, and PI3K/AKT/mTOR signalling [53]. Moreover, in the tumour microenvironment, TNF-α is one of the activators of IL-8 expression [44].

Similarly, simultaneous activation of NF-κB and HIF-1α can synergically enhance tumour development and metastasis formation [119]. Nevertheless, their effects could be strongly suppressed by the co-application of low-toxic, multi-targeting natural compounds such as curcuminoids and flavonoids with potent anti-metastatic effects [124]. For example, curcumin is a direct inhibitor of NF-κB signalling, and its application can also repress the hypoxic phenotype by targeting HSP90 and mTOR (HIF-1α stabilisation and expression) [84,125,126]. Furthermore, flavonoids inhibit various signalling pathways associated with cell migration and metastatic activity, such as MAPK, AKT, mTOR, STAT3, and/or NF-κB pathways [127]. Moreover, both types of agents display low toxicity, and their application is favourable for patients.

A promising strategy could also be based on a dual inhibitor for simultaneous targeting of mitochondrial metabolism and IL-6 signalling pathway. In a recent study, it was reported that bis-pentamethinium salts could inhibit the gp130 protein and disturb mitochondrial respiration [128]. Nevertheless, their mitochondrial uptake is too fast, and thereby their effect on IL-6 may be limited. However, their structural motif can be used as an appropriate starting point in the design of these novel dual inhibitors. It is interesting to note that suitably designed pentamethimium salts display very strong inhibition activity against dihydroorotate dehydrogenase [129], which catalyses the mitochondrial step of de novo pyrimidine synthesis (conversion of dihydroorotate to orotate) [130]. Because the enzyme prosthetic group flavin mononucleotide serves as an acceptor of a dihydroorotate electron, its inhibition can cause disturbance of mitochondrial respiration [131]. In prostate cancer cells, pentamethinium application led to the imbalance of the mitochondrial metabolism, which is strongly associated with the repression of cell migration and invasiveness [129].

Clinically used inhibitors of IL-6 signalling, such as IL-6R antibody, could be promising tools to assist classical anticancer treatment (e.g., chemotherapy and radiotherapy). For example, in the mouse model of mucoepidermoid carcinoma, tocilizumab repression of STAT3 and AKT phosphorylation caused a significant decrease in tumour growth, drug resistance, and increased overall survival [132]. Although in vitro, the tocilizumab application did not display any cytotoxicity in mucoepidermoid carcinoma cells. It decreased the subpopulation of cancer stem cells (ALDH^high^CD44^high^) and in vivo repressed paclitaxel-related induction of this cancer stem cell phenotype. Tocilizumab could also be a prospective agent in combination therapy for treating radiotherapy patients. Matsuoka et al. reported that higher IL-6 levels could be observed in squamous cell carcinoma cells and tissue samples from squamous cell carcinoma patients [133]. In squamous cell carcinoma cells, IL-6 supports cell survival via STAT3 and nuclear factor erythroid 2-related factor 2 signalling. Based on that, it was hypothesised that tocilizumab application could strongly enhance the radiosensitivity of the tested cells. Moreover, in order to increase the efficacy of treatment, therapeutic strategies can also simultaneously target various levels of IL-6 signalling. Dual application of tocilizumab and stattic significantly repressed IL-6-induced expression of vimentin and VEGF and downregulation of E-cadherin in DU-145 prostate cancer [134]. This phenomenon was associated with a substantial decrease in cell viability, colony formation, and migratory and invasive capacity against single-target inhibition.

The above-mentioned facts strongly suggest that targeting IL-6 signalling could greatly enhance the commonly used anticancer modalities. Nevertheless, numerous clinical trials are requested for the validation of this hypothesis and the optimisation of possible therapeutic strategies.

## 7. IL-6 Signalling in Selected Cancer Types

### 7.1. Head and Neck Squamous Cell Carcinoma

HNSCC is the most common cancer type of the head and neck region. Epidemiologically, HNSCC is one of the top ten most common cancer types worldwide. It affects the epithelium of the oral cavity, pharynx, and larynx [135]. It is most commonly associated with the use of tobacco products, alcohol consumption, poor oral hygiene or infection, namely by human papillomaviruses (HPV) [135,136]. Population-based screening for HNSCC has proven ineffective, as most patients do not present with pre-malignant symptoms [135]. While 5-year survival rates for patients with early-stage HNSCC are good, around 80%, this figure rapidly drops with cancer spread to lymph nodes down to 40%. Further, the survival with metastatic spread falls to 20% only [137]. IL-6 has been shown to be one of the molecules whose levels correlate with HNSCC progression and patient survival [24].

In HNSCC, a specialised subpopulation of cells, cancer stem cells (CSC), localise to a perivascular niche, and this convenient proximity to an organ’s vasculature most likely allows subsequent migration and intravasation into blood vessels [25,138]. CSC have unlimited potential for proliferation and self-renewal, thus being able to perpetuate the growth of HNSCC [25,136]. The tumorigenic potential of CSC correlated with IL-6 levels, as confirmed in both mice transplanted with HNSCC and in tissue sections from HNSCC patients [25]. Kim and colleagues generated CRISPR/Cas9 IL-6 knockout endothelial cells, which were co-implanted with UM-SCC-22B cells to form xenograft tumours and then implanted into mouse models. Slowed tumour growth was observed with IL-6 knockout endothelial cells when compared to the control, suggesting that secreted endothelial IL-6 advanced the migratory phenotype of the cancer cells. Furthermore, cancer stem cell migration in vitro was also reduced when treated with antiIL-6 antibodies or tocilizumab, and cultures had a smaller fraction of cancer stem cells, a key piece of data helping to further describe the contribution of cancer stem cells and IL-6 in tumorigenesis and metastatic spread [138,139]. Additionally, Wang et al. found increased expression of mRNA of IL-6 and IL-6R in human tumour samples when compared to the physiological oral mucosa, with higher expression also being associated with larger tumours and more advanced histological grade [140]. Overall, data have shown that IL-6 prepares cancer stem cells, likely via a chemotactic mechanism, for the epithelial-to-mesenchymal transition (EMT), essential for the next step of the invasion-metastasis cascade [25,138].

**Figure 6:**
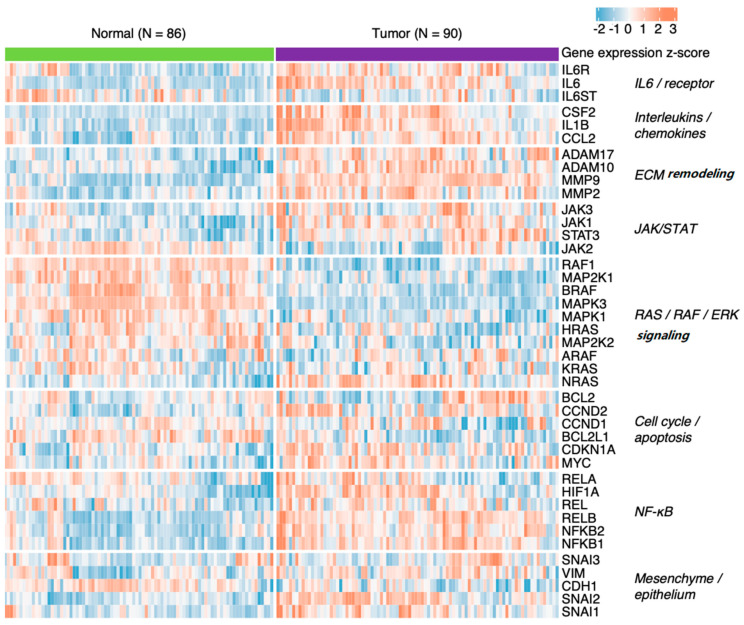
Deregulation of IL-6 signalling and its downstream targets in HNSCC tumours compared to matched samples of adjacent healthy mucosa (see Novotný et al., 2020 [141] for details).

Novotný et al. (2020) [141] suggested compensatory deregulation of the genes coding for cyclins D1 and D2 in HNSCC. Moreover, analysis of their publicly available data (ArrayExpress accession E-MTAB-8588) using the DESeq2 Bioconductor package [142] showed deregulation of IL-6 pathway components, including some of its downstream targets (Figure 6).

### 7.2. Ovarian Cancer

Ovarian cancer is the most lethal of female genital cancers because of a lack of early clinical symptoms in the patient and a lack of effective screening methods. As a result, the malignancy is usually discovered at an advanced stage, worsening the survival rate of patients. IL-6 is prevalent in the TME of ovarian cancer and, via complex signalling and response by both cancer and stromal cells, is able to promote proliferation, angiogenesis, and migration while inhibiting apoptosis [19,32,143]. The overactivation of IL-6 pathways, particularly activation of STAT3, has been implicated in the aggressiveness of ovarian cancer [144]. Activation of STAT3 by IL-6 allows expression of cell cycle-promoting proteins such as cyclin D1, D2, and c-MYC and downregulation of cyclin-dependent kinase inhibitor p21, facilitating entry into the cell cycle, thus enhancing the growth of the tumour [32,145]. Saini and co-workers observed that activated STAT3 is highly expressed in ascites-derived ovarian cancer cells (ADOCC). When transplanted into the ovarian bursa in mice, ADOCC proceeded rapidly to generate large tumours as well as extensive metastases to the liver and peritoneum. STAT3-knocked-down ADOCC failed to form metastases and resulted in slower tumour growth [144]. Using the SKOV-3 cell line and treatment with ascites fluid from three patients with advanced serous ovarian carcinoma, Kim et al. showed an increase in cellular migration and invasion in response to treatment. The effect was only seen in SKOV-3 ovarian cancer cells and not in immortalised ovarian surface epithelial cells [26]. Additionally, the ascites-treated SKOV-3 cells showed a mesenchymal phenotype with decreased levels of E-cadherin (epithelial marker) and increased levels of Snail and vimentin (mesenchymal markers) [26]. Subsequent analysis showed increased levels of IL-6 in ovarian cancer patient ascites, and treatment using anti-IL-6 antibodies showed decreased invasion and migration, decreased mesenchymal phenotype, and decreased activation of the JAK/STAT3 downstream signalling [26]. Increased invasiveness of IL-6-expressing ovarian cancer cells was also demonstrated by Wang et al. Invasiveness was evaluated based on cell proliferation, ability to invade Matrigel-coated Transwell chambers, and expression of matrix metalloproteinases 2 and 9. In A2780 (cells not expressing IL-6), highly invasive behaviour was observed after overexpression of IL-6. Compared to A2780 untransfected controls, the authors observed better anchorage-independent growth and enhanced cell migration. The effect was abrogated when IL-6-expressing SKOV-3 cells transfected with an antisense IL-6 plasmid showed decreased invasive abilities [146].

### 7.3. Breast Cancer

Breast cancer is the second leading cause of cancer-related deaths in women [147]. It was estimated that every one in eight women would develop breast cancer in their lifetime. In breast cancer, several studies have shown that patients with breast cancer have increased serum levels of IL-6. Moreover, there is evidence that IL-6 levels correlate with worse survival rates in patients, especially in those with metastatic breast cancer [14,148]. Early research in vitro demonstrated IL-6-dependent motility of human ductal carcinoma cells. Morphologically, the carcinoma cells initially had an epithelioid morphology, but upon exposure to IL-6, promptly adopted a stellate or fusiform shape. This was associated with increased motility of the cells and loss of their cell-cell junctions [149]. Recently, it was shown that IL-6-dependent repression of E-cadherin expression and weakening of adherens junctions correlates with invasiveness and metastatic potential by promoting an EMT phenotype both in in vitro experiments and mouse models [150]. Chang et al. demonstrated in mouse models that both IL-6 and downstream activated transcription factor STAT3 were present at the leading edge of breast tumours, suggesting a link between the presence of IL-6 and the invasive behaviour of the tumour itself. Indeed, Chang and colleagues proposed a feed-forward mechanism. In the suggested model, paracrine IL-6 signalling from tumour cells also activated p-STAT3 and IL-6 expression in stromal components–namely endothelial cells, CAFs (cancer-associated fibroblasts), and myeloid cells. This further functionally enhanced the IL-6/JAK/STAT3 signalling axis within the TME. This feed-forward mechanism dictated the ability of the tumour to proliferate, establish vascular supply, regulate the degree of inflammation, and determine the metastatic potential of the tumour [31]. Additional studies further proved the pivotal role that IL-6 plays in the aggressiveness of breast cancer by stimulating a stem-like phenotype in MCF-7 mammospheres, characteristic of basal-like breast carcinoma, and IL-1β-dependent expression of IL-6, which increased stemness, invasiveness, and survival in MCF-7 cells [151,152].

### 7.4. Melanoma and Cutaneous Squamous Cell Carcinoma

The incidence of melanoma has been rising around the world, despite the incidence of other cancers decreasing [153]. Melanoma is an aggressive malignant disease of multifactorial aetiology. Melanoma is often primarily resistant to various oncological therapeutical modalities, making treatment difficult. Until recently, melanoma was nearly incurable in the case of metastatic disease [154].

In a spheroid-based model using A2058 human melanoma cells, Jobe et al. showed that in cultures with conditioned media (CM) from A2058 + CAF, CAF, or normal primary fibroblasts, invasiveness increased the most in spheroids cultured in CM from the A2508 + CAF condition [34]. Upon analysing levels of IL-6, it was found that the levels in CM were increased especially in cultures of CAF or fibroblasts, suggesting that these cells are the dominant producers of IL-6 within the TME. However, the most marked increase in IL-6 was from the co-culture of A2508 or BLM cells together with CAF [34]. When anti-IL-6 antibodies were added to the cell culture, the invasive effects were significantly diminished [34]. Fibroblasts co-cultured with invasive human melanoma cell lines showed increased expression of chemokines and cytokines such as IL-1β, IL-8, and IL-6. IL-1β is thought to promote invasiveness by inducing the expression of pro-inflammatory signalling molecules such as IL-6, and subsequent siRNA silencing of IL-1β attenuated the invasiveness of the cells [33].

Weber and co-workers recently observed in a murine melanoma model that IL-6 induced CCR5 expression and thus induced potent immunosuppressive activity of MDSC in the TME [155,156]. Increasingly in the last decade, immune checkpoint blockade therapy redefined the therapeutic options to treat advanced melanoma. However, it is a great success in clinical oncology; acquired resistance and treatment-related toxicities are widespread. Hailemichael et al. suggested recently that the combination of IL-6 blockade and dual inhibition of CTLA-4 and PD-1 can overcome these issues [157]. This identifies another critical mechanism in melanoma immune response avoidance and makes IL-6 a promising target for melanoma immunotherapy.

Depner and colleagues were able to identify a possible mechanism by which IL-6 activation of stromal fibroblasts contributes to the metastatic potential of human squamous cell carcinoma (SCC) [158]. In in vitro organotypic co-cultures with human fibroblasts and a human skin carcinogenesis model (HaCaT-ras A-5RT1 cell line), IL-6 was found to activate fibroblasts and encourage progression to the tumour-associated fibroblast phenotype, activating expression of metalloproteinase-2, thus promoting the invasive capabilities [158]. In addition to paracrine signalling, exosomes produced by melanoma cells stimulated the production of IL-6 by CAFs, which improved in vitro migration of melanoma cells from the heterogeneous spheroids containing melanoma cells and CAFs in 3D collagen gels [159,160].

Taken together, these experiments demonstrate the significance of the cross-talk between stromal and tumour cells in cancers of the skin and elucidate the mechanisms by which IL-6 is able to promote invasiveness in cancers of the skin.

## 8. Conclusions

The IL-6 signalling pathway plays a significant role in cancer biology, particularly in its involvement in metastasis formation. Targeting its principal components (e.g., IL-6Rs, gp130, STAT3, NF-κB) is an intensively studied approach that is of translation potential in patients, as it can affect the course of treatment. Should targeting IL-6 be insufficient, it can be used as a complementary treatment along with chemo- and radiotherapy. Nevertheless, IL-6 signalling is not an isolated phenomenon. It must be observed as an important part of a complex system, and unlocking its full potential will very likely require either targeting IL-6 signalling at several levels or in combination with inhibition of other signalling pathways. However, numerous well-conducted studies strongly imply the remarkable potential of IL-6 signalling inhibitors, especially in metastasis suppression.

## Figures and Tables

**Table 1:** Examples studied agents for the inhibition of IL-6 signalling with an emphasis on their clinical applicability.

Name (Trade Name)	Target: Function	Status	Ref.
**Human monoclonal antibody**			
**Tocilizumab** (RoActemra)	**IL-6R**: receptor inhibition	Clinically used for rheumatoid arthritis	[47]
		Case report: Reducing IL-6-mediated cachexia	[48]
**Sarilumab** (Kevzara)	**IL-6R**: receptor inhibition	Clinically used for rheumatoid arthritis	[49]
		Under clinical trial (EMPOWER NCT04333706): triple-negative breast cancer (stage I-III, high-risk residual diseases) combination with Capecitabine	
**Siltuximab**	**IL-6**: neutralization	approved for CAR-T	[50]
**Low molecular inhibitor**			
**Bazedoxifene** (Conbriza)	Estrogen receptor modulator	Clinically used in the treatment of osteoporosis	[51]
	**gp130**: inhibitor	In vivo	[52]
	**CD40 receptor**: inhibitor	In vitro	[53]
**Tofacitinib (Xeljanz)**	JAK1/3 inhibitor	Clinically used in the treatment of moderate-severe ulcerative colitis	[54]
	JAK pathway	Clinical trial: developed malignancies lung, breast, gastric cancer, and lymphoma; rate of malignancies by 6-month intervals of tofacitinib exposure indicates rates remained stable over time	[55]
**Ruxolitinib** (Jakafi)	JAK1/2 inhibitor	Clinically used in the treatment of steroid refractory graft-versus-host disease	[56]
	JAK pathway	Clinical trials: inadequately controlled polycythaemia; decrease in thromboembolic events	[57]
**Momelotinib**	ACVR1/ALK2, JAK1 and JAK2, inhibitor	FDA accepts for the treatment of the myelofibrosis	
**Momelotinib**		Clinical trials: myelofibrosis; higher overall and leukaemia-free survival.	[58]
**Madindoline A and B**	**gp130**: inhibitor	In vitro	[59,60]
**ERBF**	**IL-6R**: blocking interaction IL-6R with IL-6, or gp130	In vivo	[61,62,63]
**Stattic**	**STAT3**: inhibition of activation	In vivo	[64]
**OPB-31121**	**STAT3**: inhibition of activation	Clinical trials: advanced colon and rectal tumours; tumour shrinkage, bad pharmacokinetic (very low blood concentration)	[65,66]
**Galiellalactone**	**STAT3**: inhibition of DNA binding	prostatectomy samples: reduction IL-6 induced AR signalling	[67]
		In vivo	[68]
**GPB730**	**STAT3**: inhibition of DNA binding	In vivo	[69]
**OPB-51602**	**STAT3**: activation of aggregation	Clinical trials: refractory haematological malignancies; no clear therapeutic response was observed	[70]
**Ixazomib**	**NF-κB**: inhibition of ubiquitin-proteasome pathway leading to loss of NF-κB activity	Clinical trials: Relapsed or Refractory Cutaneous or Peripheral T-cell Lymphomas; reduction in NF-κB activation and subsequently GATA-3 expression in the biopsy specimens	[71]
**Theofyline** (Elixophyllin, Elixophylline, Pulmophylline, Quibron-T, Theo-24, Theolair, Uniphyl)	phosphodiesterase inhibitor, adenosine receptor blocker, and histone deacetylase activator	Clinically used in chronic obstructive pulmonary disease and asthma	[72,73]
	**NF-κB**: inhibition of activation	In vitro	[74]
**Rapamycin** (Sirolimus, Rapamur)	**mTOR**: inhibitor	Clinically used immunosuppressive therapy	[75]
		Clinical trials: acute myelogenous leukaemia; no effects on the composite complete remission rate	[76]
	**IL-6, TNF-α and IL-1β**: decrease cytokine level	In vivo	[77]
**Zotarolimus**	**IL-1β, TNF-α, IL-6 and NF-κB**: decrease cytokine level and NF-KB activity	In vivo	[78]
**NSAIDs** (e.g., celecoxib, aspirin, ibuprofen, naproxen, meloxicam)	**cyclooxygenase inhibitors**	in a broad spectrum of conditions; Analgetic, antipyretics, in rheumatic diseases	[79]
**Celecoxib**	**IL-6**: decrease expression by COX-2 inhibition	Clinical trials: former-smokers; bronchoscopy samples (reduction IL-6 and Ki-67 expression)	[80]
**Food supplements**			
**Curcumin**	**IL-6**: decrease expression	Clinical trials: patients with solid tumour; decrease in plasma level of IL-6, TNF-a, TGF-b, substance P, hs-CRP, CGRP and MCP-1, increase patient quality life	[81]
	**STAT3**: decrease activity	In vivo	[82]
	**NF-κB**: activity and expression	Clinical trials: advanced pancreatic cancer; peripheral blood mononuclear cells (decrease in expression of NF-κB, STAT-3 and COX-2) decrease in serum cytokine levels (IL-6, IL-8, IL-10, and IL-1 receptor antagonists)	[83]
	**HIF-1α**: decrease expression and activity	In vitro and In vivo	[84,85]
**Epigallocatechin-3-gallate**	**NF-κB**: decrease expression	Clinical trial: subjects with a high risk of colorectal cancer; lover expression of the NF-κB and DNMT1	[86]
	**STAT3**: inhibition of activation	Molecular assay and In vitro	[87]

## Data Availability

Not applicable.
